# NeAT: a Nonlinear Analysis Toolbox for Neuroimaging

**DOI:** 10.1007/s12021-020-09456-w

**Published:** 2020-03-24

**Authors:** Adrià Casamitjana, Verónica Vilaplana, Santi Puch, Asier Aduriz, Carlos López, Grégory Operto, Raffaele Cacciaglia, Carles Falcón, José Luis Molinuevo, Juan Domingo Gispert, for the Alzheimer’s Disease Neuroimaging Initiative

**Affiliations:** 1 Department of Signal Theory and Communications, Universitat Politècnica de Catalunya (UPC), Barcelona, Spain; 2 QMENTA, Barcelona, Spain; 3 Vilynx, Barcelona, Spain; 4 BarcelonaBeta Brain Research Center (BBRC), Pasqual Maragall Foundation, Barcelona, Spain; 5 Alzheimer’s Disease and Other Cognitive Disorders Unit, Hospital Clínic, Institut d’Investigacions Biomèdiques August Pi i Sunyer (IDIBAPS), Barcelona, Spain; 6 CIBER Fragilidad y Envejecimiento Saludable (CIBERFES), Madrid, Spain; 7 Universitat Pompeu Fabra, Barcelona, Spain; 8 IMIM (Hospital del Mar Medical Research Institute), Barcelona, Spain; 9 Centro de Investigación Biomédica en Red de Bioingeniería, Biomateriales y Nanomedicina (CIBER-BBN), Madrid, Spain

**Keywords:** nonlinear, neuroimaging, GLM, GAM, SVR, Alzheimer's disease, inference, APOE

## Abstract

NeAT is a modular, flexible and user-friendly neuroimaging analysis toolbox for modeling linear and nonlinear effects overcoming the limitations of the standard neuroimaging methods which are solely based on linear models. NeAT provides a wide range of statistical and machine learning non-linear methods for model estimation, several metrics based on curve fitting and complexity for model inference and a graphical user interface (GUI) for visualization of results. We illustrate its usefulness on two study cases where non-linear effects have been previously established. Firstly, we study the nonlinear effects of Alzheimer’s disease on brain morphology (volume and cortical thickness). Secondly, we analyze the effect of the apolipoprotein APOE-ε4 genotype on brain aging and its interaction with age. NeAT is fully documented and publicly distributed at https://imatge-upc.github.io/neat-tool/.

## Introduction

The increase of computational power and advances in neuroimaging acquisition that enable faster scans and provide multiple image contrasts and modalities has motivated the development of complex modeling techniques for imaging data. An armoury of neuroimaging analysis tools is available to the neuroscientific community, whose ultimate goal is to conduct statistical tests to identify significant effects in the images without any a priori hypothesis on the location or extent of these effects. In the literature, analysis at different levels of brain morphometry are found, involving voxel-based (Penny et al. 2011), surface-based (Fischl 2012) or boundary-based analysis (Freeborough and Fox 1997).

Irrespective of their particular characteristics, the vast majority of them perform statistical inference upon different implementations of the General Linear Model (GLM). GLM has been shown to be flexible enough for conducting most of the typical statistical analysis (Friston et al. 1994). However, it has a rather limited capability to model nonlinear effects. In this regard, it is worth noting that linear models have been reported not to be sufficient to fully describe cerebral structural variation with cognitive decline (Samtani et al. 2012; Mendiondo et al. 2000) or associated to pathological progression in neurodegenerative disease (Villemagne et al. 2013; Insel et al. 2017; Insel et al. 2015; Sabuncu et al. 2011; Schuff et al. 2012; Bateman et al. 2012; Gispert et al. 2015). Moreover, many relevant confounders in neuroimaging are shown to be better described by nonlinear processes, such as the impact of aging on cognitive decline (Kornak et al. 2018) or gray-matter volume (Fjell et al. 2013). Under the GLM, the modeling of non-linear effects is limited to using polynomial expansion or transforming the variables of interest to linearize their effects. However, such approximations are suboptimal (Fjell et al. 2010; Vinke et al. 2018; Ziegler et al. 2012). On the other hand, a wide range of non-linear modeling methods have been developed but specific implementations that enable the unbiased analysis of neuroimaging data are lacking (Breeze et al. 2012).

In this work, we describe a new analytic toolbox which is able to model nonlinear effects on brain scansat the voxel-wise level as well as for surface data. We pool together several nonlinear parametric models, provide different model comparison strategies and implement a graphical user interface (GUI) for visualization purposes. In the following sections we briefly describe the main functionalities of the toolbox and illustrate its features with two studies: (i) nonlinear atrophy patterns across the Alzheimer’s disease continuum defined as a function of cerebrospinal fluid (CSF) biomarkers (Gispert et al. 2015) and (ii) the effects of apolipoprotein E4 genotype on brain aging, a risk factor to develop sporadic Alzheimer’s disease (AD) (Cacciaglia et al. 2018).

## Material and Methods

A general overview of the tool operatibility and its options and functionalities are introduced in this section. A detailed mathematical description of the curve fitting methods and statistical inference metrics is provided, even though the reader is encouraged to read the original sources for a more deep understanding of such methods. More instructions on how to download and use the tool can be found in https://imatge-upc.github.io/neat-tool/.

### NeAT Overview

The NeAT toolbox is a modular and easy-to-use toolbox for the analysis of non-linear effects on medical brain images. Several curve fitting methods are used to model the relationship between certain factors (e.g: age, disease phenotype, genotype) and pre-processed scans. Any image modality that has been spatially normalized and is ready for voxelwise analysis can be submitted to NeAT (e.g: Normalized VBM modulated images (Ashburner and Friston 2000) or FDG PET scans (Frackowiak et al. 1980)), as well as cortical thickness data resulting from Freesurfer processing (Fischl 2012). Those methods may include multiple covariates (factors) that can be split into confounder factors and variables of interest by using contrasts. A simple preprocessing step allows to orthogonalize, orthonormalize or simply normalize all covariates. A wide range of metrics can be used to assess the goodness of fit of each model. Statistical inference also allows the use of contrasts on modeling factors. The embedded 3D visualization GUI provides a unified and interactive environment to visualize both 3D statistical inference maps and the estimated curve at each voxel.

**Fig. 1:**
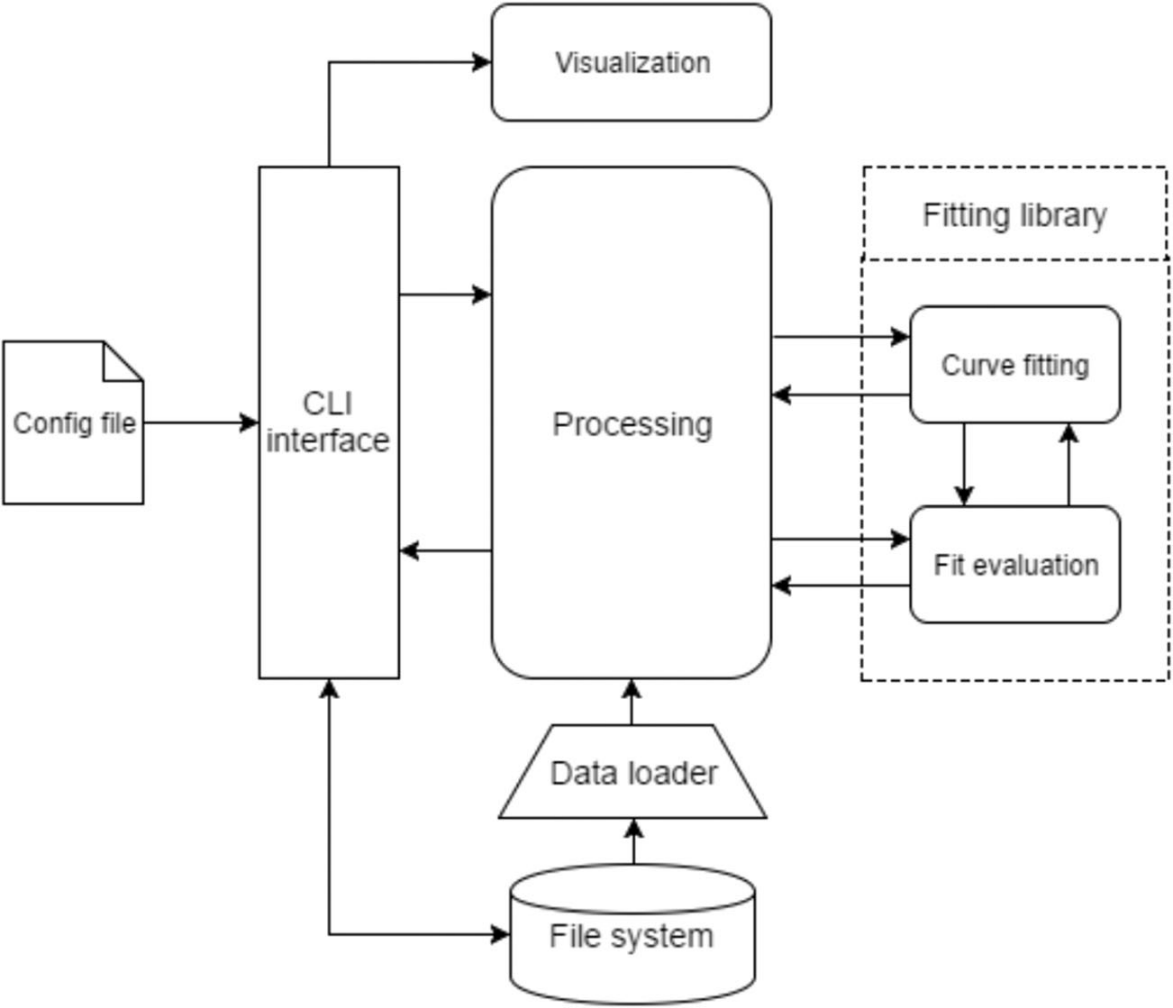
Toolbox pipeline. The *Processing* module govern the interaction between all other libraries that will be explained through the manuscript

A high-level overview of the toolbox pipeline is provided in Fig. 1. It consists of several interdependent modules connected through a Processing library that performs serialization between functionalities. Each other module (Curve Fitting, Fit Evaluation and Visualization) is designed separately using abstract classes that facilitate both continuous adaptation and possible extensions of the toolbox. A description of each module/ functionality is detailed in the following sections.

### Model Estimation

The model estimation step (Curve Fitting module) is in charge of finding a parametric function of several explanatory variables that best fits the observations in terms of maximizing a quality metric or minimizing a loss function. Different specification of the latter two give rise to different models or fitters. To analyze the basics of each fitter, we consider the regression model

$$ Y=f(X)+E $$

where $Y=\left[{y}^1,{y}^2,\dots, {y}^N\ \right]\in {\mathfrak{R}}^{LxN}$ are the N dependent observations (e.g. number of voxels), $X=\left[{x}_1,{x}_2,\dots, {x}_M\ \right]\in {\mathfrak{R}}^{LxM}$ are the M independent factors constant for all observations, $f(X)\in {\mathfrak{R}}^{LxN}$ is the fitted curve and $E=\left[{e}^1,{e}^2,\dots, {e}^N\ \right]\in {\mathfrak{R}}^{LxN}$ is the estimation noise. Each input variable (*y*^*i*^, *x*_1_, …, *x*_*M*_) is an L-dimensional vector corresponding to different measures (e.g. different subjects) of the same magnitude. Each covariate can be independently entered and the overall estimated model is found by adding up the contribution of each one:

$$ \hat{y}={\sum}_{m=1}^M{f}_m\left({x}_m\right) $$

being f_m_(x_m_) the associated curve fitting method for each covariate. The available methods.

are detailed below. All observations are processed in chunks and fitted independently.

(*y*^*i*^ = *f*^*i*^(*X*) + *e*^*i*^, where *i* represents each observation). Data processing (normalization and orthogonalization) techniques are optionally prepended to the overall analysis.

In this toolbox we consider the general framework that splits explanatory variables into.

variables of interest (predictor variables) and confounder factors (corrector variables) as explained in Henson and Penny (2003). The goal of this scheme is to deduct confounder effects on the dependent variables to isolate the main effects of the variables of interest we want to analyze. This paradigm is widely used in neuroimaging: for example, using age (corrector).

as confounder variable when analyzing the effect of Alzheimer’s disease (predictor).

on hippocampus volume (observation or dependent variable). Concretely, we split the initial space *S*, defined by all explanatory variables *X*, into two subspaces: predictor (*S*_*P*_) and corrector (*S*_*C*_) subspaces of dimensions *M*_*C*_ and *M*_*P*_, respectively (*M* = *M*_*C*_ + *M*_*P*_). The predictor subspace is defined using a contrast matrix *C*, described by *X*_*P*_ = *X* · *C*, and its model is defined as ${\hat{Y}}_P={f}_P\left({X}_P\right)$. On the other hand, the corrector subspace is built using a null-contrast matrix (orthogonal to the contrast matrix), *C*_0_ = *I* − *C* · *C*^#^, where *C*^#^ is the pseudoinverse of *C*. Hence, the corrector subspace is described by *X*_*C*_ = *XC*_0_, and its model is defined as ${\hat{Y}}_C={f}_C\left({X}_C\right)$. Even though C and C_0_ are orthogonal, both subspaces are orthogonal only if the columns of X are orthogonal.

We model the contribution of each subspace on the overall effect using an additive model $Y={\hat{Y}}_P+{\hat{Y}}_C+E$, fitting first the corrector model (*Y* = *f*_*C*_(*X*_*C*_) + *E*_*C*_ ) on the observations and then the predictor model (*E*_*C*_ = *f*_*P*_(*X*_*P*_) + *E*) on the residuals. Since the fitting is done separately, both corrector and predictor functions, *f*_*C*_ and *f*_*P*_ can be any nonlinear model implemented in the toolbox. Note that each corrector and predictor variables can be modeled using different curve fitting methods:

$$ \hat{Y}={\hat{Y}}_C+{\hat{Y}}_P={f}_C\left({X}_C\right)+{f}_P\left({X}_P\right)={\sum}_{c=1}^{M_C}{f}_c\left({x}_c\right)+{\sum}_{p=1}^{M_P}{f}_p\left({x}_p\right) $$

Baseline curve fitting methods implemented in the toolbox are: (i) GLM, (ii) GAM and (iii) SVR. Each subspace (predictor and corrector) can be modeled by any of these techniques. While the first two methods model each dimension independently, the third allows for interactions between different dimensions.

#### General Linear Model: GLM

The General Linear Model (Christensen 2011) is the extension of multiple regression models to the case of multiple observations. The effect of each factor is independently analyzed without accounting for interactions between them. The model reads as follows:

$$ y=f(X)+e= X\beta +e, $$

where *β* are the model parameters and *e* is the error of the model. GLM optimization involves minimizing the mean squared error ${\left\Vert\ e\ \right\Vert}_2^2$ between data points and the fitted curve. Nonlinear relationships can be modeled in the GLM framework by using a polynomial basis expansion of each regressor. The total number of degrees of freedom is the number of covariates in the analysis (df = M), including each basis expansion if used.

#### Generalized Additive Model: GAM

A Generalized Additive Model (Hastie 2017) is an extension of additive models (AM) to the case of multiple observations. In GAM, each observation depends on unknown smooth functions of each covariate:

$$ y=f(X)+e={f}_1\left({x}_1\right)+{f}_2\left({x}_2\right)+\dots +{f}_M\left({x}_M\right)+e. $$

In the context of this toolbox, *f*_*i*_ refer to parametric smooth functions, called smoothers, that are iteratively estimated using the backfitting algorithm (Breiman and Friedman 1985) to minimize the mean squared error ${\left\Vert\ e\ \right\Vert}_2^2$. If linear or polynomial smoothers are used, GAM is equivalent to GLM. Other smoothers available are B-splines or natural splines, implemented using the Patsy library (https://patsy.readthedocs.io/en/latest/). The total number of degrees of freedom is the sum of degrees of freedom of each smoother *df* = *df*_1_ + *df*_2_ + … + *df*_*M*_. For a linear smoother, the number of degrees of freedom is one (*df*_*i*_ = 1), for polynomial smoother, the number of degrees of freedom is the polynomial order (*df*_*i*_ = *d*) and for splines-based smoothers, the number of degrees of freedom is an input parameter set by the user.

#### Support Vector Regression: SVR

Support Vector Regression (Drucker et al. 1997) is a multivariate method that inherently accounts for interactions between covariates unlike GLM or GAM, that only account for the additive effect between covariates. In SVR the goal is to find a function *f*(*X*) that has at most *ε*-deviation from the observations and is as smooth as possible. However, since the *ε*-deviation constraint might not be feasible, a hyperparameter C controls the balance between smoothness and errors greater than *ε*. SVR is a linear method in the parameters with a closed form solution. To introduce nonlinearities, SVR uses the *kernel trick* which implicitly transforms the inputs to a higher dimensional feature space by only specifying their inner product, i.e. the kernel function *k*(*x*^*i*^, *x*^*j*^) =  < *φ*(*x*^*i*^), *φ*(*x*^*j*^)>, where *x*^*i*^ and *x*^*j*^ are two feature vectors from different observations. Once estimated, the overall model is parameterized using parameters *β* as follows

$$ y=f(X)+e={\sum}_{l=0}^{L-1}k\left({x}^l,x\right){\beta}_l+e, $$

where *x*^*l*^ are all data points used to fit the model and *x* is any feature vector. Two kernel functions are implemented in this toolbox using the scikit-learn library (Pedregosa, 2011): polynomial and the radial basis function (RBF) defined as *k*(*x*^*l*^, *x*) =  *exp* ( − *γ*‖ *x*^*l*^ − *x*‖^2^, where *γ* is a hyperparameter defining the width of the kernel. The total number of degrees of freedom depends on the kernel used and it is based on the solution proposed in Dinuzzo et al. (2007).

##### Hyperparameter Search

SVR relies on the election of several hyperparameters: *ε*and *C* for the general solution and kernel related hyperparameters, such as *γ*in RBF kernels. The hyperparameter values can be automatically determined by a grid search on the hyperparameter space (Hsu et al. 2003). This method consists of several steps: (i) sample H different value combinations from the hyperparameter space using one of the sampling strategies provided in this toolbox: random or deterministic sampling with linear or logarithmic scale, (ii) fit a subset *G*of the observations on all H hyperparameter combinations and (iii) select the hyperparameter combination that minimizes the metric of interest, *t*_*i*_, on the subset *G* (*T* = ∑_*i* ∈ *G*_*t*_*i*_). The available metrics are: (i) minimum squared error, (ii) F-test goodness of fit and (iii) Mallows’s C_p_ statistic (James et al. 2013). To avoid selection bias, this procedure is iterated varying the selected subset *G* of observations. Larger subset sizes provide better hyperparameter estimations but increasing time and memory requirements, due to the intensive search performed. However, we allow parallelization of the second step and further iterations of the algorithm. To account for the great between subject variability of medical images the voxelwise metric values are weighted by the inverse of the variance (1/*σ*_*i*_) of each observation $\left(\hat{t_i}={t}_i/{\sigma}_i,\kern0.75em \hat{T}={\sum}_{i\in G}\hat{t_i}\right)$. Moreover, due to the vast amount of background voxels, only those with minimum variance (*σ*_*min*_) can be included in the subset of observations.

### Statistical Inference

Statistical maps evaluating the goodness of fit and penalizing by the complexity of the model can be computed for each of the fitting methods presented in section 2.2. To this purpose, several metrics are available in the tool:

- **Minimum squared error (MSE) and Coefficient of determination (*R***^**2**^**)**: these two metrics evaluate the predictive power of the model without penalizing for its complexity.
  1 $$ MSE={\left\Vert\ y-f(X)\ \right\Vert}_2^2 $$
  2 $$ {R}^2=1-\frac{SS_{res}}{SS_y},\kern1.25em {SS}_{res}={\left\Vert\ y-f(X)\ \right\Vert}_2^2,\kern1.25em {SS}_y={\left\Vert\ y-\underset{\_}{y}\right\Vert}_2^2 $$

where $\underset{\_}{y}=\frac{1}{N}{\sum}_{i=0}^{N-1}{y}^i$ is the mean of the observations.

- **Akaike Information Criterion (AIC):** the AIC criteria (Sakamoto et al. 1986) is founded on information theory. It is useful for model comparison as it provides a trade-off between the quality or goodness of fit and the complexity of the model, which is proportional to the number of parameters.
  3 $$ AIC=2k-2 LLR,\kern1.25em LLR=-\frac{N}{2}\left(\mathit{\log}\left(2\pi \cdotp MSE\right)+1\right) $$

where *k* is the total number of parameters and LLR is the log likelihood ratio.

- **F-test:** the F-test is a statistical test following an F-distribution under its null-hypothesis. In the context of this toolbox, it evaluates whether the variance of the full model (correctors and predictors) is significantly lower than the variance of the restricted model (only correctors). Under the null-hypothesis, the full-model does not provide any significantly better fit than the restricted model, resulting an F-statistic with (df_full_, df_restricted_) degrees of freedom and the corresponding *p* value. Rejection of the null hypothesis is based upon the p value.
  $$ {f}_{score}=\frac{SS_{res}-{SS}_{full}}{SS_{full}}\frac{N-{df}_{full}}{df_{full}-{df}_{res tricted}} $$
  $$ {p}_{value}=1-F\left({f}_{score},{df}_{res},{df}_{full}\right) $$

where ${SS}_{res}={\left\Vert y-{f}_c\left({X}_c\right)\right\Vert}_2^2$, ${SS}_{full}={\left\Vert y-{f}_C\left({X}_C\right)-{f}_P\left({X}_P\right)\right\Vert}_2^2$, and *F*(*x*, *d*_1_, *d*_2_)is the F-distribution.

- **Penalized Residual Sum of Squares (PRSS), Variance-Normalized PRSS (VNPRSS)**: PRSS is introduced in this toolbox as another evaluation metric that accounts for the goodness of fit and penalizes the model complexity. However, differently from other metrics, complexity is not computed with the degrees of freedom but using the curve shape itself. Hence, a complex model such as SVR with Gaussian kernel that provides a linear curve will penalize as much as the GLM. VNPRSS is an adaptation of PRSS for data with high-variability, like medical images, and penalizes each error term by the inverse of the observations variance.
  4 $$ PRSS= MSE+\gamma \cdotp {c}_{abruptness},\kern1.25em {c}_{abruptness}=\int f^{\prime\prime }(X)\  dx\kern0.5em $$
  5 $$ VNPRSS=\frac{PRSS}{c_{variance}},\kern1.25em {c}_{variance}={\left\Vert \kern0.5em f(X)-\underset{\_}{y}\ \right\Vert}_2^2 $$

### Post-hoc Analysis

The NeAT toolbox provides several functionalities for post-hoc analysis of the generated curves and statistical maps. Different model comparison strategies and a curve clustering algorithm are presented in what follows.

#### Model Comparison

In order to compare L statistical maps generated using different fitting models we combine them into a single statistical map providing different information:

- **Diff-map** (L = 2): it provides the difference between maps, being useful for quantitative detection of differences between L = 2 fitting models.
- **ABSdiff-map** (L = 2): it provides the absolute difference between maps, being useful for quantitative detection of differences between L = 2 fitting models.
- **SE-map** (L = 2): it provides the squared difference between maps, being useful for quantitative detection of differences between L = 2 fitting models.
- **RGB-map** (L = 3): it places each map in a different color channel. It might be useful to compare the intersection of several fitting models showing agreement and disagreement among them.
- **Best-map** (L > 1): it computes the best fitting model at each voxel. It might be useful for model localization in the brain.

#### Clustering

We incorporate a curve clustering functionality (Jacques and Preda 2014) for extracting distinct pattens of brain data variation with respect covariates of interest. In that sense, we provide a scalable and non-parametric algorithm that is able to explore similarities and dissimilarities of the fitted curves across the brain and group them in a total of *N*_*C*_ clusters.

We adopted the hierarchical clustering framework (Murtagh and Legendre 2014) implemented in scikit-learn (Pedregosa et al. 2011). It is a bottom-up approach where initially each curve defines its own cluster. Next, pairs of clusters are successively merged according to a certain similarity metric and a linkage criterion. As a similarity metric, we use a weighted sum of distances:

6 $$ SD\left(x,y\right)={\sum}_{i=0}^{N_D-1}{w}_n{d}_n\left(x,y\right) $$

where (*x*, *y*)are two different curves, *d*_*n*_ is the Euclidean distance between the *n*^*th*^ discrete derivative of each curve, *w*_*n*_ is the weight of each derivative to the total similarity metric and *N*_*D*_ is the total number of derivatives used. In our implementation, we fix *N*_*D*_ = 3 and ***w***^***T***^ ***=*** [**0.2**, **0.8**, **0.2**]. As a linkage criterion, we use the average distance between all possible pairs of elements of both clusters

7 $$ L\left(A,B\right)=\frac{1}{\mid A\mid \cdotp \mid B\mid }{\sum}_{a\in A}{\sum}_{b\in B} SD\left(a,b\right) $$

where (*A*, *B*) are two different clusters, (| *A*| , | *B*| ) are the cardinalities of the clusters and (*a*, *b*) represent a curve from each cluster. Hence, at each step of the hierarchy, the two clusters that minimize the linkage criterion are combined. The algorithm stops when it reaches *N*_*C*_clusters (a parameter predefined by the user).

Please note that there is not a single optimal value for the number of clusters (N_c_). Hence, we include a functionality to plot the variance between and within clusters as well as the silhouette coefficient (SC) metric (Rousseeuw 1987) that can be used to assess the optimal number of clusters for the analysis as a trade-off between within and between cluster distance.

### Visualization

This toolbox provides *show-curves* and *show-data-distribution* functionalities and a graphical user interface (GUI) for visualization purposes. The *show-curves* is a command line functionality that reads either the voxel coordinates in mm (x,y,z) for voxel-based morphometry (VBM) analysis, or the vertex number (x) for surface-based morphometry (SBM) analysis, both referenced to the template specified in the configuration file. The *show-data-distribution* functionality allows the user to visualize the input data distribution (observations, residuals, covariates) using different types of plots: univariate and bivariate densities, boxplots and a categorical boxplots.

#### Graphical User Interface (GUI)

An interactive visualization GUI for 3D volumes (VBM) is provided for further analysis of the results. It allows to load 3D overlays over a template and visualize the generated curves for one or several fitting models of interest. Overlays must have the same extension as specified in the configuration file and can be either generated by the tool (e.g: statistical maps, model comparison maps or clustering maps) or external (e.g: brain structure atlases). Simultaneously, it shows the three orthogonal planes (axial, coronal and sagittal) and the curve of the corresponding voxel. Inspection of the overall brain and associated curves can be done online using the cursor in an interactive way.

Due to long rendering times, for visualization of 2D surfaces (2D) we recommend using other visualization software (e.g: FreeSurfer) in parallel with the *show-curves* functionality.

### NeAT Specifications

NeAT toolbox uses a configuration file to specify experiment related options such as input/output files or experiment parameters. The overall analysis pipeline (*model estimation, statistical inference, visualization*) is split into smaller steps using different scripts. A command line interface (CLI) is used for communication between the toolbox and the user, allowing to run the scripts and set specific parameters for the analysis (e.g. which fitting module to use as model estimator).

The toolbox input files consist of covariates and images. Input covariates need to be stored in a spreadsheet either *.csv* or *.xls* extension. Input images can be either preprocessed using voxel-based morphometry (VBM) or surface-based morphometry (SBM): nifti formats (*.nii/.nii.gz*), the Massachusetts General Hospital formats (*.mgh/.mgz*) and measurements of cortical thickness (*.thickness*) and surface area (*.area*) can be used in the tool.

Each analysis step of the global pipeline generates different output files saved under the directory specified in the configuration file. Statistical maps are saved using the same extension as input files allowing compatibility with other neuroimaging packages (e.g: visualization software). As a programming language, Python (version 3.6) is used due to its object-oriented programming paradigm that provides flexibility in toolboxes with increasing size and complexity. Moreover, Python is becoming progressively popular in the neuroimaging field with growing scientific libraries (e.g: scipy (Jones et al. 2014)), neuroimaging (e.g: nibabel (Brett et al. 2016)) or machine learning toolkits (e.g: scikit-learn (Pedregosa et al. 2011)).

## Validation Results and Discussion

To exemplify NeAT’s main functionality, it has been applied to three case studies where non-linear behaviour of neuroimaging data has been described previously.

### Case Study 1: Atrophy Patterns across the Alzheimer’s Disease Continuum

#### Voxelwise Volumetric Analysis

Nonlinear volumetric changes in gray matter across the Alzheimer’s disease (AD) continuum have been described (Gispert et al. 2015). In this report, nonlinearity is modeled using GLM with a 3rd-order polynomial basis expansion, and the relevance of linear against higher-order predictors was compared. Here, we use NeAT to fit several nonlinear models to the same dataset in order to statistically compare them.

In brief, study participants were enrolled in a single-cohort study from the Alzheimer’s Disease and Other Cognitive Disorders Unit in Hospital Clinic of Barcelona (HCB). The cohort comprises 129 subjects (62 controls, 18 preclinical AD, 28 mild cognitive impairment (MCI) due to AD and 21 diagnosed AD) that underwent an MRI scan, registered to a common space, and a CSF lumbar puncture. The AD continuum is defined biologically by the AD-CSF index (Molinuevo et al. 2013) which combines CSF biomarkers into a single indicator that determines the position of each subject along the AD continuum. For further details on both MRI processing and CSF acquisition, refer to Gispert et al. (2015).

Following the standard procedure of splitting covariates into confounding factors and predictors, we fit a corrector GLM model using sex and a second order polynomial expansion of age. We use AD-CSF index as the predictor variable fitting several models to the GMv corrected observations: (i) GLM with third order polynomial expansion, (ii) GAM using b-splines as smoothing function (iii) SVR using third order polynomial kernel and (iv) SVR using Gaussian kernel. We use an F-test to statistically compare all predictor models. Statistical significance was set to *p* < 0.001 uncorrected for multiple comparisons with a cluster-extent threshold of 100 voxels.

**Fig. 2:**
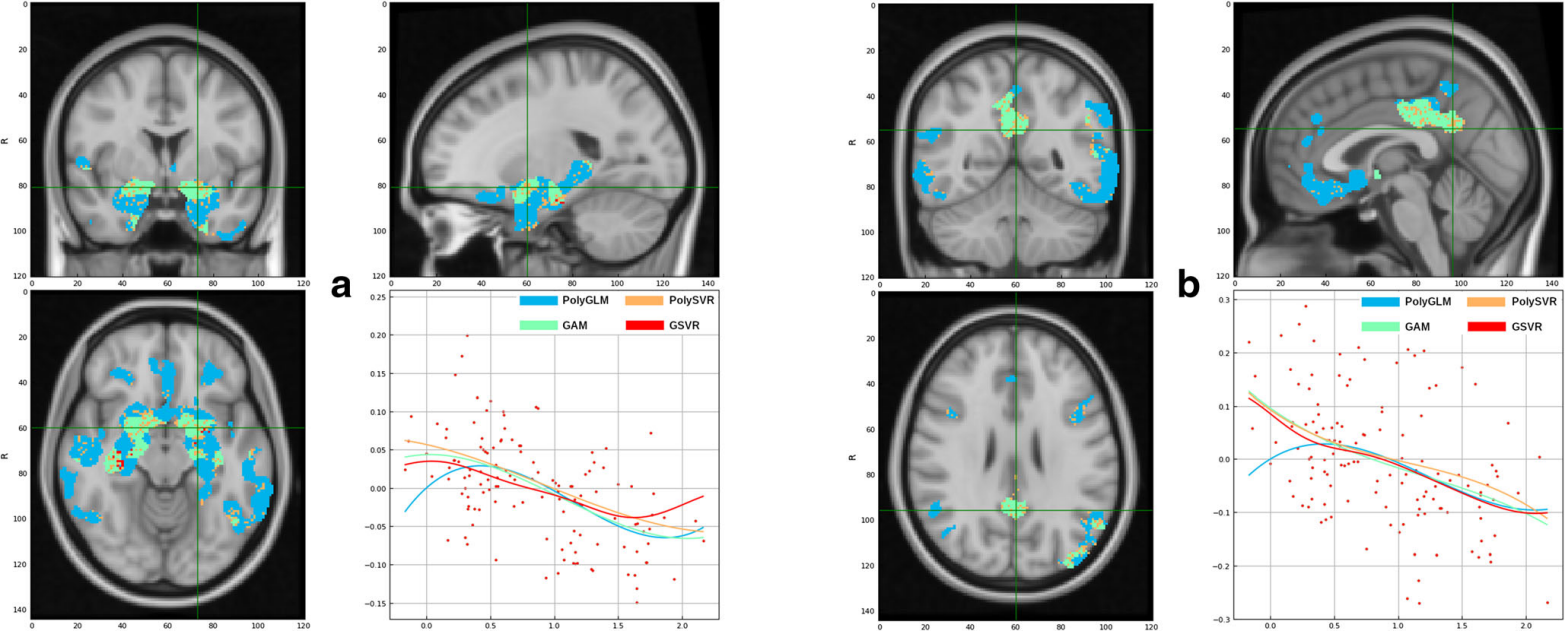
Comparison between different curve fitting models: third order polynomial expansion of GLM (blue), B-splines GAM (green), SVR with polynomial kernel (yellow) and SVR with Gaussian kernel (red). The best-map is used for statistical comparison, showing the best (in terms of F-test) model among all four models with statistical significance using uncorrected *p* < 0.001 separately for each model. Estimated curves show the variation of gray matter volume (y-axis) and AD-CSF index (x-axis). Based on CSF amyloid-beta and tau levels, the AD-CSF index measures biomarker progression using a single index normalized between 0 (no altered biomarkers) and 2 (full AD-like alteration) (Molinuevo, 2013). The figure on (A) corresponds to the left hippocampus and the figure on (B) corresponds to the right precuneus

Figure 2 shows a few examples of the visualization GUI using the *best-map* option to compare the aforementioned fitting methods. Results using GLM with polynomial basis expansion are coherent with the ones found in Gispert et al. (2015). However, better goodness-of-fit can be achieved using nonlinear models in NeAT and, in particular, GAM seems to better fit extreme values. There is a high overlap between second order polynomial expansion of GLM, GAM with b-splines and SVR with polynomial and Gaussian kernels. Due to the low numbers of degrees of freedom used, GLM and GAM appear to be the most relevant models across the brain. On the other hand, using a Gaussian kernel on SVR employ higher number of degrees of freedom and its relevance is restricted at the center of typical AD subcortical regions (e.g: hippocampus and amygdala).

**Fig. 3:**
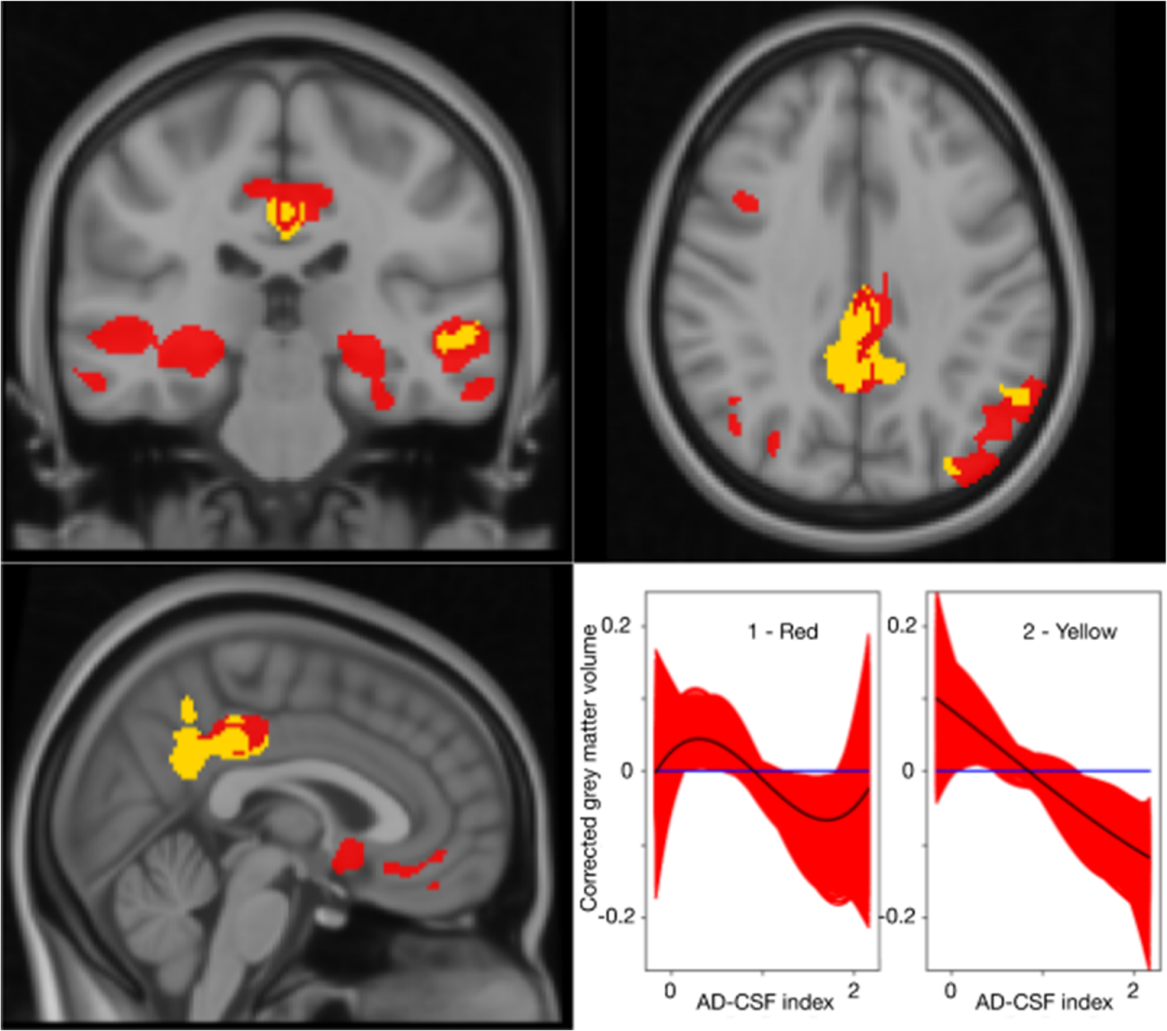
Curve clustering algorithm run on relevant atrophy patterns along the AD-CSF index using GAM fitting. The number of clusters is set to N_C_ = 6. On the left, we show the relevant voxels color-coded to describe the association of each voxel with each cluster. On the right, we show all curves associated to each cluster (red) and their respective centroid (black)

Further analysis of the results can be done using the clustering functionality of the tool. Using the GAM model, we look for regions with similar atrophy patterns along the AD continuum. We compute the silhouette for a large number of clusters and end up with an optimal number of *N*_*C*_ = 2 clusters, with a silhouette average value of *S* = 0.32. In fig. 3 we show the results with the curves for each cluster and their associated brain regions. We can clearly distinguish two different patterns: a linear pattern involving region such as the precuneus or the cingulate cortex while another non-linear pattern group other regions such as the middle temporal or hippocampus.

#### ROI Cortical Thickness Analysis

To further validate the toolbox we perform a cortical thickness analysis along the Alzheimer’s continuum. Global cortical thinning is known for Alzheimer’s disease patients even though the evolution may vary temporally along the continuum and spatially across the brain. Hence, nonlinear models are flexible to model such variability.

**Fig. 4:**
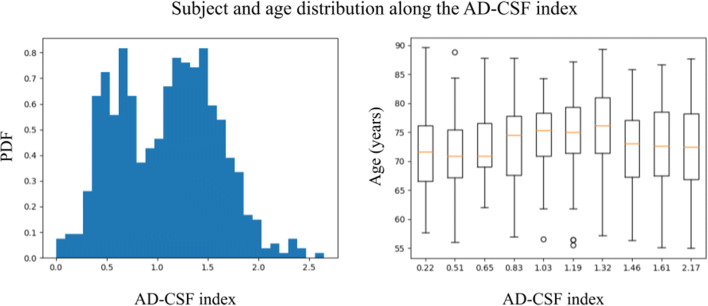
Subject distribution (left) and age distribution (right) along the AD-CSF index of the subset of ADNI used in the analysis. For the subject distribution we compute the histogram while for the age distribution we show a boxplot splitting the AD-CSF index into deciles

In this analysis we use publicly available data from the Alzheimer’s Disease Neuroimaging Initiative (ADNI, http://adni.loni.usc.edu/). We use baseline average cortical thickness for each of the K_ROI_ = 68 ROIs using the Desikan-Killiany atlas (Desikan et al. 2006) and CSF biomarkers measurements from a total of 610 subjects. We use a sex and a second order polynomial expansion of age as correctors. From CSF biomarkers we use Aβ and tau values to construct the AD-CSF index (Molinuevo et al. 2013) as the predictor. In fig. 4 we show the distribution of subjects and its related age along the AD-CSF index. Following ADNI guidelines, 191 subjects labeled as cognitively unimpaired, 284 subjects were labeled as having mild cognitive impairment and 135 subjects were diagnosed with dementia.

**Fig. 5:**
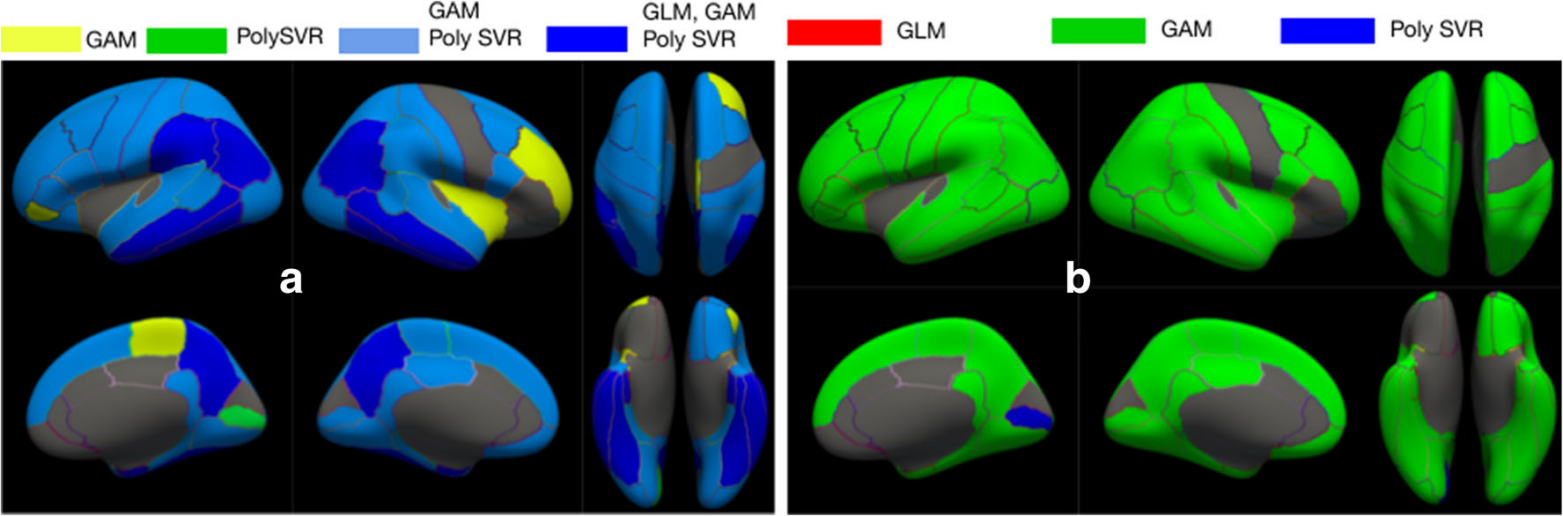
Statistical comparison maps between three different curve fitting methods (GLM, GAM and SVR with polynomial kernel). We use an RGB map (A) to show regions relevant for each method with the following legend: yellow (only GAM) green (only SVR), light blue (GAM and SVR) and dark blue (GLM, GAM, SVR). We use the best map (B) to show the method with best statistical inference metrics with the following legend: red (GLM), green (GAM), blue (SVR)

**Fig. 6:**
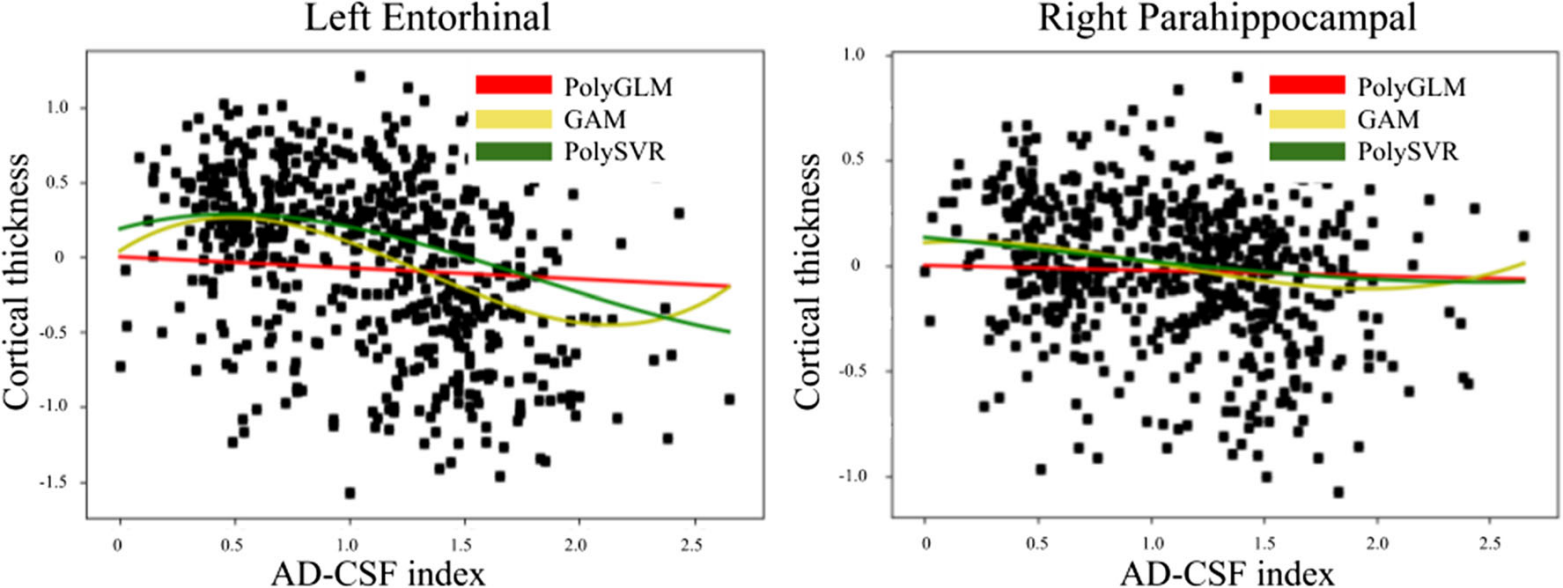
Generated curves for the evolution of cortical thickness of the left entorhinal (left) and the right parahippocampal (right) regions. For each ROI we use a linear (GLM) and two nonlinear (GAM and SVR with polynomial kernel) models. All three are statistically relevant for the left entorhinal while only the two nonlinear models appear to be relevant for the right hippocampal

We compare linear and non-linear models, being the latter more statistically significant across the brain (figs. 5, 6). In fig. 5 we show statistical inference maps comparing three different fitting methods: (a) GLM, (b) GAM and (c) SVR with polynomial kernel. For each method we compute and F-test with statistical significance *p* < 0.001 uncorrected. Using a best map we see that GAM method has generally better inference metrics while using the RGB map we see that the linear method missed many regions outside the temporal lobe. Finally, Fig. 6 shows the fitted curves for the left entorhinal and the right hippocampus. Similar effects on the extreme values as the ones described in the previous dataset can be observed in Fig. 6 with parametric fitting, which are much alleviated with other non-linear fitting methods.

### Case Study 2: Effects of *APOE*-ε4 in Brain Aging

The ε4 allele of the apolipoprotein E (*APOE*) gene is the strongest genetic risk factor for AD. *APOE* is polymorphic and contains three different alleles referred as *APOE*-ε2, −ε3 and -ε4 coding three different isoforms and six different genotypes. Here, we apply NeAT to analyze the interaction between *APOE*-ε4 allele load and age on the brain morphology of middle-aged cognitively unimpaired individuals, thus expanding previously published results in Cacciaglia et al. (2018). The ALFA (ALzheimer’s and FAmilies) cohort presented in Molinuevo et al. (2016) was used for this purpose, involving 533 subjects that underwent *APOE* genotyping and an MRI scan. For statistical analysis, participants were pooled according to the *APOE*-ε4 allele load: 65 homozygotes (HO) that have *APOE*-genotype with 2 copies of the *APOE*-ε4 allele, 207 heterozygotes (HE) with a single copy of the *APOE*-ε4 allele and 261 non-carriers (NC).

#### *APOE* Genotype Effects on Brain Morphology in Normal Aging

**Fig. 7:**
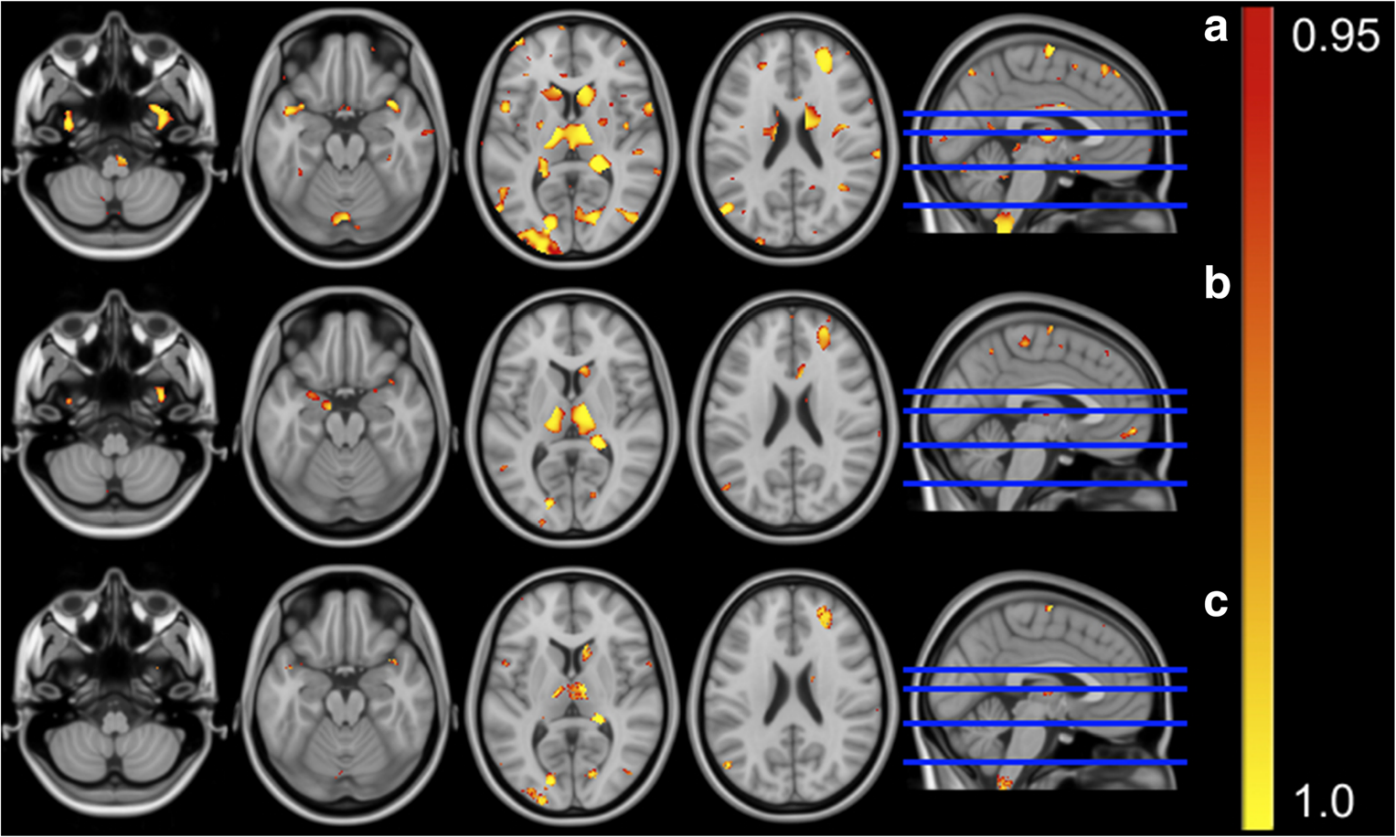
Statistical inference using volumetric data and different curve fitting modules: using GLM (A), using GAM (B) and using SVR with a polynomial kernel (C). For visualization purposes, statistical significance threshold is set to *p* < 0.05 uncorrected

In this case study, we replicate the results of Cacciaglia et al. (2018) with respect to the *APOE* genotype effects on brain morphology with NeAT and use it to expand previously described non-linear effects. The baseline model consists of three dummy variables characterizing each genotype (NC, HE, HO) defining the number of ε4 alleles. Sex, years of education, total intracranial volume and linear and quadratic expansions of age were included as covariates. Due to the reported interactions (ten Kate et al. 2016) of *APOE* status and age, we fit the model with the interaction terms *APOE*x *age* and *APOE*x *age*^2^. We apply the contrast [−1,0,1] on dummy variables indicating *APOE-ε4* allele load, defining an additive model that predicts incremental/decremental effects of *APOE-ε4* homozygotes. Results using the linear model are shown in fig. 7(A), replicating the findings in Cacciaglia et al. (2018). The use of the tool allowed us to study non-linear effects of the genotype. Concretely, in fig. 7b and c we show results using GAM and SVR with polynomial kernel models, respectively. Smaller effects are observed and only relevant effects are found in regions such as bilateral thalamus, right hippocampus, right superior frontal and small cluster around the right caudate and the left middle occipital. Nonlinear modeling fail behind linear modeling of *APOE-ε4* count, probably because it is a categorical (C = 3) predictor. Hence, due to higher degrees of freedom used in GAM and SVR, only larger significant values survive the used threshold.

**Fig. 8:**
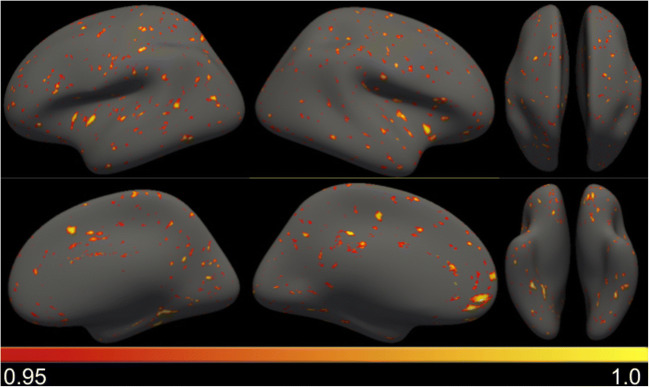
Statistical inference using cortical thickness data and GLM. For visualization purposes, statistical significance threshold is set to *p* < 0.05 uncorrected

Using the tool, we could also study the *APOE* genotype effects in cortical thickness data. In fig. 8, we show the results on different surface views using the GLM model. In this case, even smaller effects are found being statistically relevant (*p* < 0.05) in small clusters across the brain, specially in regions such as the insular cortex and fusiform.

#### Interaction between *APOE* Genotype and Age in Normal Aging Population

In this second part, we investigate the interaction between *APOE* genotype and age on brain morphology. For this purpose, we model each *APOE* genotype separately to find their associated curves and generate a goodness-of-fit metric using the F-test. Statistical inference threshold is set to *p* < 0.001. We perform post-hoc analysis combining statistical maps into an RGB-map that sums up the results of all three *APOE*-genotype models: we place each model (NC, HE, HO) in each R, G, B channel, respectively. Volumetric and cortical thickness analyses were performed but no significant results were found with the later.

**Fig. 9:**
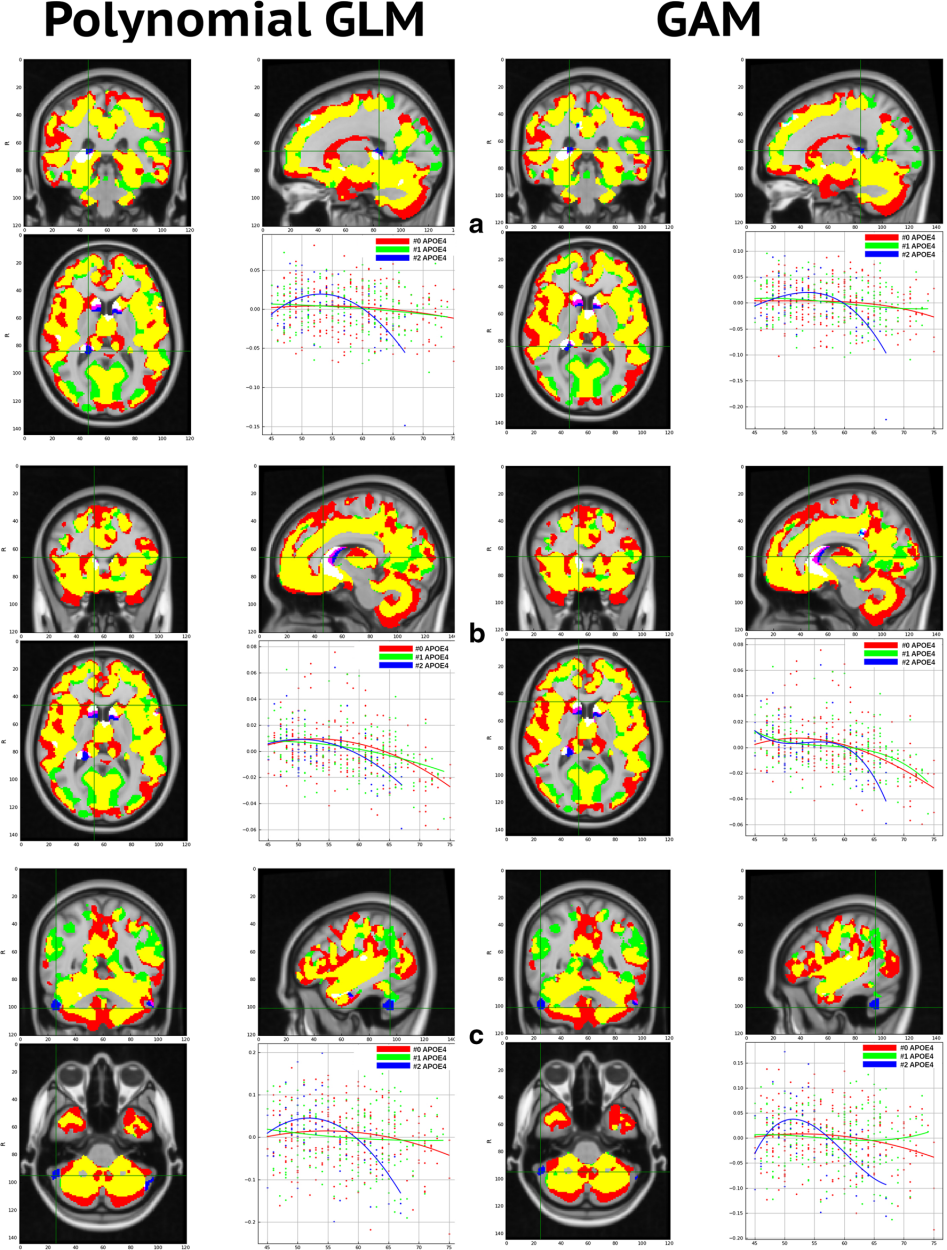
Interaction between age and the APOE-ε4 genotype using second order polynomial expansion of GLM (left) and B-splines GAM (right). Three different regions are shown at each row: (A) right hippocampus, (B) right caudate and (C) right cerebellar crus. Statistical analysis using F-test and uncorrected *p* < 0.001 threshold with cluster size of 100 voxels. Statistical comparison using the RGB map, where R corresponds to 0 copies of the allele, G to 1 copy and B to 2 copies. Other colors are any possible combination of them, meaning that are relevant for more than one APOE genotype

In fig. 9, we show the RGB-map and the associated curves of regions corresponding to significant effect of age on brain morphology of homozygotes *APOE*-e4 carriers (see section 3.2.1): right hippocampus, right caudate and right cerebellar crus. We present two different curve fitting models: using polynomial expansion of second order of the GLM on the left and B-splines GAM on the right.

**Fig. 10:**
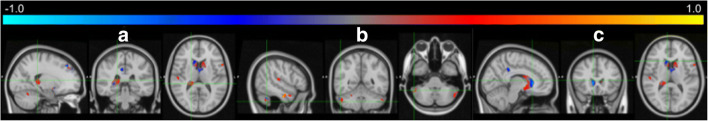
Differences between statistical maps of the HE model using GLM and GAM at different brain ROIs: right hippocampus (A), right caudate (B) and right cerebellar crus (C). A positive (negative) value indicates that GAM (GLM) is statistically better using the f-test metric

Clearly, relevant regions for the HO group show nonlinear relationship between age and voxel intensities. Statistical and *RGB-maps* present analogous results on polynomial expansion of GLM and GAM analysis. The right hippocampus and the right cerebellar crus follow a quadratic curve with age similar to GLM fitting. HO subjects show an earlier decreasing of GMv in both regions compared to NC and HE around their fifties with an initial volumetric increase in middle-aged individuals, more pronounced in the cerebellum, again replicating the results in Cacciaglia et al. (2018). On the other hand, GMv volume on the right caudate appears to decrease at the sixth decade for all *APOE* genotypes but decaying faster for HO subjects. Due to the non-quadratic behaviour of the right caudate, it appears to be better modeled with GAM, as shown in fig. 10.

## Conclusions

In this paper, we present NeAT; a tool for non-linear analysis of neuroimaging data at the voxel or surface levels and illustrate its functionality in three case studies where a nonlinear behavior of brain morphology was previously described. NeAT is a modular, flexible and user-friendly toolbox that provides advanced curve fitting methods for voxelwise and surface-based modeling and different metrics for statistical inference of the results. Visualization features are available, such as an interactive GUI that shows statistical maps together with the resulting fitted curves. Finally, post-hoc analysis functionalities such as model comparison (e.g: linear vs. non-linear) or a curve clustering algorithm that show similar fittings across the brain are available. Altogether, NeAT constitutes a complementary tool for the standard processing of non-linear associations between neuroimaging data and a set of factors (e.g: age, environmental factors, disease, genetics or demographics) at the voxel and surface levels.

### Future Work

the potential of NeAT is expected to expand as it will grow. At the short term, the expansion of the tool to ROI-based analysis is granted. Moreover, a longitudinal analysis module might be interesting due to the increasing number of cohorts with longitudinal follow-up visits. Seemingly, the integration of fMRI modality should be considered in future revisions of the toolbox. Other statistical methods, such as Partial Least Squares (PLS) or Canonical Correlation Analysis (CCA) can be incorporated for multivariate effects modeling. Finally, other curve fitting models (e.g: based on neural networks) can be designed and implemented.

## Information Sharing Statement

The source code of the presented method is freely available for non-commercial use from https://imatge-upc.github.io/neat-tool/.
